# Geriatric Patients’ Experiences with Dietary Patterns and Medication Behaviours in Type 2 Diabetes: A Qualitative Exploration

**DOI:** 10.21315/mjms-11-2025-847

**Published:** 2026-06-30

**Authors:** Mika Tri Kumala Swandari, Zakky Cholisoh, Hidayah Karuniawati

**Affiliations:** 1 Faculty of Pharmacy, Universitas Muhammadiyah Surakarta, Sukoharjo, Central Java 57162, Indonesia; 2 Faculty of Pharmacy, Science and Technology, Universitas Al-Irsyad, Cilacap, Central Java 53223, Indonesia

**Keywords:** dietary patterns, medication behaviour, geriatrics, type 2 diabetes mellitus

## Abstract

**Background:**

Type 2 diabetes mellitus (T2DM) in geriatric patients requires effective self-management, particularly regarding dietary habits and medication behaviour, to achieve optimal blood sugar control. This study aimed to examine the self-management practices of older adults with T2DM, focusing on their dietary habits and medication use behaviour.

**Methods:**

This qualitative study used semi-structured interviews involving geriatric patients with T2DM recruited from the Department of Endocrinology at a secondary referral teaching hospital. Data were thematically analysed using a predefined interview framework.

**Results:**

The findings indicated that most geriatric patients with T2DM could adopt healthy eating patterns, such as reducing rice portions, substituting carbohydrates with alternative sources and increasing the consumption of vegetables and low-fat proteins. Strategies to control restricted food intake included self-reminders, avoiding visual exposure and spiritual approaches. Regarding medication behaviour, non-adherence was identified due to forgetfulness, medication burden and the use of non-prescription drugs, such as herbal medicines. These findings emphasise the importance of continuous patient education and social support to promote independent diabetes self-management among older adults.

**Conclusion:**

This study revealed that geriatric patients with T2DM adopted various strategies for managing their diet and medication behaviour, including portion control, healthy food selection, spiritual approaches and the use of herbal products. Most of the participants showed awareness and adaptability in pursuing a healthy lifestyle. However, persistent challenges were identified, such as ingrained habits, forgetfulness in medication adherence and feeling a medication burden. Continuous educational and social support is essential to enhance the effectiveness of self-management in the holistic care of older adults with diabetes.

## Introduction

Type 2 diabetes mellitus (T2DM) is a chronic metabolic disorder that is highly prevalent among older adults (1). Globally, an estimated 463 million individuals are living with diabetes, including approximately 111 million people over 65 years of age, placing this population at increased risk for complications and functional decline. Diabetes currently ranks as the 11th leading cause of disability worldwide (2).

Cognitive, physical and psychological impairments are the major contributors to treatment failure among older adults with T2DM (3). These functional limitations reduce their ability to manage dietary intake and adhere to medication, thereby increasing the risk of diabetes-related complications (4). Inadequate self-management worsens the risk of comorbid geriatric syndromes (4).

Inconsistent adherence to dietary and pharmacological regimens, such as missed doses or improper medication usage, increases the risk of side effects (5), potentially leading to serious complications, such as amputations, myocardial infarctions and fatal falls (5). Age-related factors, such as loss of appetite and gastrointestinal symptoms, further complicate dietary management (6). In addition, the use of non-prescribed substances, such as herbal products, is common among older adults and can result in harmful drug interactions (7).

This study explores dietary and medication management behaviours among geriatric patients with T2DM at a regional public hospital in Central Java, Indonesia, which serves as a diabetes referral centre. It aims to identify key challenges and barriers to achieving optimal self-management. Currently, no study in Indonesia has specifically examined these combined behaviours in older adults, particularly in a hospital-based referral setting. The findings will provide evidence-based insights for healthcare providers in developing targeted education, counselling and intervention programmes to improve adherence, minimise complications and enhance quality of life.

## Methods

### Design

In this phenomenological qualitative study, the first author conducted in-depth interviews using a descriptive qualitative approach. Colaizzi’s seven-step analysis was used to analyse and categorise textual data, considering the individual cultural and contextual effects on the phenomenon (8).

### Participants and Sampling

Geriatric patients over 60 years of age with T2DM for more than one year were recruited from the hospital’s pharmacy unit for interviews. The participants were selected through coordination between the research team, the hospital director and the pharmacists. The interviews focused on the participants’ self-management behaviours and their experiences in managing diabetes.

The inclusion criteria were as follows: patients aged over 60 years with a diagnosis of T2DM for at least one year, able to read and write and willing to provide informed consent for participation and audio recording. Patients with a history of stroke or communication impairments, such as difficulties in vision, hearing or speech, as assessed by the interviewers, were excluded from the study.

Purposive sampling was applied to ensure the recruitment of participants with diverse backgrounds and varying disease durations. Recruitment took place from 11 November to 26 November 2024.

### Interview Framework and Data Collection

The interview guide framework covered the participants’ daily diet, dietary restrictions, medication adherence, feelings of treatment burden and use of complementary medicine and was developed based on expert review and validation. Various aspects of self-management were considered with the flexibility to explore emerging topics during the interviews. The interviewer obtained basic demographic and clinical information from each participant, including age, gender, duration of diabetes and occupation. A pilot test involving 10 participants was conducted, and their data were excluded from the final analysis. Interviews were scheduled in advance and conducted in the hospital counselling room after written informed consent was obtained. All sessions were audio-recorded, supplemented by field notes and lasted 30 to 90 min. The semi-structured interview guide is provided in Appendix 1.

### Ethics Statement

This study received ethical approval from the Health Research Ethics Committee, Faculty of Health Sciences, Universitas Muhammadiyah Surakarta (Ref: 270/KEPK-FIK/VI/2024).

### Data Analysis

All interview data were anonymised and transcribed verbatim. The data were analysed manually using a thematic approach. The process began with repeated readings of the transcripts to ensure familiarity with the content. This was followed by line-by-line coding, conducted independently by the first two authors (Mika Tri Kumala Swandari and Hidayah Karuniawati). Codes were subsequently organised into themes and sub-themes through iterative discussions between these two authors. Disagreements were resolved through consultation with the third author (Zakky Cholisoh), who has expertise in diabetes management. This process continued until consensus was achieved on the final list of themes and sub-themes.

## Results

### Participants’ Characteristics

Identifying demographic characteristics is essential for understanding T2DM self-management in geriatric patients. Table 1 outlines the key participant demographic data, providing an important context for understanding the study population.

Table 1 shows that most of the participants were male, aged 61 to 81 years, had a normal body mass index and were no longer employed. The majority were married, had a regular income below USD 113, lived 24.05 km to 32.00 km from the hospital and generally attended appointments alone. Most reported having controlled blood sugar levels.

### Key Thematic Outcomes of Data Analysis

#### Dietary Patterns

Interviews on dietary practices among geriatric patients with T2DM identified two primary themes: daily food intake and the control of restricted foods. For clarity and conciseness, the findings from both themes are integrated and summarised in Table 2.

The following quotations illustrate the participants’ usual daily food choices and their approaches to managing dietary restrictions. The participants described various meal patterns, with rice, cassava and vegetables frequently mentioned as staple foods. One participant stated, “In the morning, *oyek* (dried cassava rice) or sweet potato; at noon, rice; in the evening, rice” (Male, 61 years old, N8). Another participant explained, “Sea fish, vegetables, papaya leaves for salad, cassava leaves, without coconut milk” (Male, 64 years old, N12).

Several participants detailed the composition of their meals. One participant noted, “Three meals daily consisting of a vegetable stir-fry in the morning, tamarind vegetable soup without added sugar at noon and tempeh (fermented soybean cake) with only the egg white in the evening” (Male, 72 years old, N13). Another participant stated, “Tea with honey in the morning, rice at noon and rice again in the evening” (Male, 80 years old, N14).

Some participants consumed a wider variety of carbohydrate sources. As one participant shared, “Cassava chunks, mashed sweet potatoes, mashed cassava, boiled sweet potatoes, a small portion of rice, cold rice and if still hungry, add some meat and leafy greens” (Female, 62 years old, N16). Others expressed a more flexible approach to eating. According to one participant, “I eat as usual. If there is food that is to my liking, I just eat” (Male, 73 years old, N11).

The second theme highlights how the participants manage and interpret dietary restrictions in their daily lives. The following excerpts illustrate their perceptions, motivations and challenges in adhering to dietary guidelines. The participants expressed a variety of strategies and personal beliefs. One participant shared, “Our principle is that we had consumed it in the past when we were still healthy, so it does not matter if we do not consume it now. That is the principle” (Male, 64 years old, N1). Another participant explained, “Yes, I spend my time doing several activities, such as learning to read the Qur’an or engaging in other things” (Male, 75 years old, N18).

One participant expressed concern about glycaemic control, stating, “Yes, I am afraid that my blood sugar might rise whenever I want to eat… so I try to stay motivated and focus on recovery” (Female, 62 years old, N2). Others used more straightforward avoidance strategies. As one participant noted, “Avoid looking at foods and drinks that are prohibited” (Male, 64 years old, N9).

#### Medication Behaviour

Treatment adherence issues among geriatric patients with T2DM were categorised into three main themes: forgetting to take medication (due to travel, sleep or negligence), feelings of treatment burden (related to long-term use and concerns about side effects) and continued use of traditional remedies (*jamu*). These findings, which represent barriers to achieving optimal glycaemic control, are summarised in Table 3.

Forgetting to take medication was often linked to lifestyle factors, physical states or personal habits. One participant explained, “The reason for forgetting is frequent travelling and falling asleep” (Male, 62 years old, N10). Another participant admitted that improvement in symptoms sometimes led to non-adherence, “Sometimes when I feel better, I do not take the medicine” (Male, 70 years old, N20). Similarly, one participant noted, “I often forget and sometimes feel careless” (Female, 66 years old, N5).

Feelings of treatment burden emerged as another key issue, which is particularly related to the perception of taking medications continuously over the long term. One participant stated, “I often feel burdened by the continuous need to take medication. It feels like I am taking it all the time. So once in a while, I have cold beverages” (Male, 64 years old, N12). Concerns about potential side effects were also evident, as expressed by one participant, “Actually, I am concerned that the medication may affect my kidneys—this is the side effect of the medicine” (Female, 66 years old, N5). Another participant emphasised the perceived overuse of medication, “Yes, taking too many medications hehe (laughs)” (Female, 62 years old, N2).

The following quotes illustrate the participants’ continued use of traditional remedies, such as herbal drinks (*jamu*), which are often self-prepared from natural ingredients. One participant stated, “Yes, I take it with palm sugar along with *temulawak* (Javanese turmeric) and similar ingredients” (Male, 72 years old, N13). Another participant shared, “Mostly *kunir asem* drink (turmeric and tamarind concoction) that I prepare myself” (Female, 66 years old, N5). Some participants also consumed other herbal-based preparations, as one explained, “Mostly herbal. If I want to, I drink *daun kelor* (moringa leaves)” (Female, 61 years old, N4).

## Discussion

### Dietary Patterns

Based on the participants’ interviews, the findings on the daily food menu were diverse. The participants continued to consume rice but in smaller portions and supplemented their carbohydrate intake with alternatives, such as processed cassava leaves and fermented cassava (*tape*). Most of the participants enjoyed consuming green vegetables and fruits, while their protein needs were mainly met through eggs, sea fish, tofu and *tempeh*, typically prepared by boiling or grilling. However, a small number of participants reported no changes in their general daily food menu, although they continued to consume nutritious foods. These findings are in accordance with previous research, which has shown that maintaining a healthy diet is essential for diabetes management, but geriatric patients with diabetes often have inadequate dietary habits (9).

Maintaining a healthy diet is one of the essential aspects of T2DM treatment, particularly in geriatric patients (10). The interviews showed that most of the participants were able to adapt to a healthy diet pattern, although the older people generally faced challenges such as their old habits and physical limitations (11).

The findings showed that most of the participants reduced their rice portions and substituted them with complex carbohydrate sources, such as cassava tuber and sweet potatoes, which have a lower glycaemic index and a higher fibre content, thus helping to control blood sugar levels (12). They also consumed fruits and green vegetables, which are low in natural sugar, high in fibre and beneficial for glycaemic control and cardiovascular health, especially in the elderly with diabetes (13).

Foods such as white eggs, sea fish, tofu and *tempeh* provide the protein needs of diabetic diets, supporting tissue repair and overall physiological function (14). Most of the participants recognised the importance of choosing healthy, low-fat protein sources to maintain health and control diabetes, as their meals tended to be low in fat and processed through healthy methods, such as boiling or grilling (15). However, one participant still showed uncontrolled eating habits due to long-established psychological, social and dietary influences. This is consistent with other studies (16, 17), thus indicating the need for an understanding-based approach and social support to help geriatric patients implement a healthy diet (18).

Overall, the participants made an effort to maintain a healthy diet, although some still had difficulty changing old habits. Continuous education and support are important to help geriatric patients with diabetes achieve optimal glycaemic control and prevent complications (19).

Controlling dietary restrictions is essential for older adults with T2DM to prevent fluctuations in blood sugar levels. The interviews showed that the participants used various strategies, such as recalling their previous consumption when they were healthy, being aware of the risk of an increase in blood sugar, avoiding visual exposure to restricted foods and tasting only small amounts. A proper diet is key to the overall management of diabetes (20). The results of the interviews showed that the participants had different ways of controlling abstinence from food and drink consumption, reflecting their awareness of the importance of dietary regulation in managing diabetes.

One of the principles applied by the participants was “having already eaten it when they were healthy (non-diabetics),” in which they recalled their healthy period to resist the urge to consume restricted foods. This principle reflects self-efficacy and coping strategies in T2DM patients, in which the higher the self-efficacy, the more effective the coping strategies (21). This shows that awareness and self-confidence in managing health are important in maintaining healthy eating behaviours and controlling diabetes overall.

In addition, the participants applied the principle of “remembering having diabetes mellitus” as a reminder of the potential impact of consumption on blood sugar levels. This approach reflects high self-awareness and is an important strategy in preventing impulsive eating behaviours that can interfere with diabetes control (22).

Self-reminder strategies, such as the principle of “remembering having diabetes mellitus,” are important in the self-management of geriatric patients with T2DM. This awareness encourages their dietary choices and helps them avoid foods that risk increasing blood sugar levels. Endra Cita et al. (22) revealed the importance of self-care in treating T2DM. An awareness of health conditions and effective self-management can improve patients’ quality of life and help them control their blood sugar levels (22).

Some participants applied a “not seen” strategy to avoid the temptation of restricted foods. Avoiding visual stimuli helps them control cravings, thus showing awareness and self-regulation in dietary choices. Margawati et al. (23) found that watching *mukbang* videos can stimulate increased food intake among students. Visual exposure to food activates the brain’s feeding centre, which increases the desire to eat (23). In addition, Amalia et al. (24) found that exposure to culinary content on social media can affect adolescents’ eating behaviour. Visual exposure to food on these platforms has been shown to increase desire and affect food choices (24). These findings support the notion that avoiding the visual stimuli of restricted foods can help reduce the urge to consume items that are not aligned with the recommended diet for patients with T2DM.

The results of the interviews showed that some participants managed the consumption of restricted foods and beverages through fasting and physical restraint. These strategies include sleeping, practising patience, engaging in positive activities and reading the Qur’an. This approach reflects spiritual engagement and adaptive coping strategies that help distract their attention from food cravings while strengthening self-control in diabetes management (25).

Some participants adopted the principle of “reducing or limiting consumption” as a form of self-control. They still enjoyed the foods they liked, but in limited quantities to maintain blood sugar levels. This strategy shows a flexible but still controlled adjustment of eating behaviour.

Overall, the strategies used by the participants reflected an awareness of the importance of dietary management in geriatric patients with T2DM. Despite various challenges, they were able to adapt and make healthier eating choices. Supporting self-management through nutritional education and motivation from healthcare providers is essential, and it is necessary to involve the role of family, social environment and work to improve the success of diabetes management in old age (26).

### Medication Behaviour

Forgetfulness in adhering to prescribed regimens was a common issue among geriatric patients with T2DM. The interview findings indicated that 47.62% of the participants admitted to having forgotten to take their diabetes medication. The reasons for forgetting to take medication were diverse, primarily due to busyness and sleepiness. Some participants stated that they skipped medication due to laziness or because they perceived themselves as healthy. Forgetfulness is a common cause of medication non-adherence among geriatric patients with T2DM (27). Srikartika et al. (28) found that forgetting to take medication (77.3%) was the leading cause of medication non-adherence in diabetic patients.

Daily busyness and irregular sleep patterns were identified as major contributors to forgetfulness in medication adherence among geriatric patients. Some participants also reported discontinuing their medication because they felt better and perceived that they no longer needed it. This suggests that they had no bothersome symptoms, despite the importance of regular medication for optimal diabetes control. This condition emphasises the value of continuous diabetes education, particularly in geriatric patients with physical or cognitive impairments (28).

Geriatric patients with T2DM are at risk of long-term complications, such as damage to vital organs (kidneys, eyes and heart), which can worsen their health condition if they do not control their blood sugar levels (29). Non-adherence to medication can result in dangerous blood sugar fluctuations and an increased risk of diabetes complications (30). As a result, healthcare providers should implement strategies to minimise medication forgetfulness. For instance, using technology-based reminders such as alarms or apps, keeping a diary and involving one’s family support the reinforcement of adherence (31). Better counselling and management approaches should be applied for geriatric patients to reduce treatment forgetfulness and maintain optimal diabetes treatment (32).

The regular daily consumption of medication causes geriatric patients to feel a medication burden and affects noncompliance with diabetes mellitus medication intake. Based on the interview results, 11 out of 21 participants reported feeling burdened by taking diabetes medication. The main reasons included continuous medication intake, fear of medication side effects, unpleasant taste after consumption and the perception of taking excessive amounts of medication. This sense of burden is a key factor contributing to noncompliance with diabetes treatment.

This feeling of burden can be the reason for noncompliance in taking medication, risking poor blood sugar control and increasing the risk of diabetes complications. Many geriatric patients believe that excessive medication intake or its bitter taste diminishes their quality of life, increasing the likelihood of skipped doses or unintentional treatment discontinuation. This indicates the need for more patient-friendly medication management strategies, including finding treatment methods that are easier to use and reducing side effects.

The results showed that feeling burdened in taking medication can increase the risk of patient non-adherence to medication (33). Long-term treatment often contributes to medication burden due to the requirement for continuous medication intake. In addition, non-adherence to diabetes medication is particularly dangerous because it can lead to uncontrolled blood sugar fluctuations and increase the long-term risk of cardiovascular, ocular and renal complications. Therefore, healthcare providers should provide comprehensive support to encourage patients to remain compliant with their medications.

To overcome this medication burden, strategies such as reminder systems, personalised treatment approaches and continuing education about the long-term benefits and importance of therapy can be effective (33, 34). In some cases, alternative treatment options, such as more convenient medication regimens or technology-based solutions, can help reduce the medication burden and improve adherence to diabetes therapy (35, 36). Thus, a comprehensive and understanding-based approach to the physical and psychological components influencing treatment behaviour is essential to overcome medication burden and improve adherence in geriatric patients with T2DM (37, 38).

Patients with T2DM must take medication regularly, as prescribed by their doctor, to achieve treatment therapy outcomes. The use of over-the-counter medications remains a challenge for hospitals in ensuring successful T2DM treatment. Based on the interviews, most of the participants (13 of 21) reported self-initiated use of over-the-counter or herbal medicines. The various herbs used included turmeric, Javanese turmeric and moringa leaves. This finding suggests that many geriatric T2DM patients are looking for alternative treatments to help manage their disease. Herbal or natural products are often perceived as safer alternatives to synthetic medications, which can have adverse effects (39).

The use of herbal medicine as an adjunct therapy for T2DM is not new. Herbal medicine is already known as traditional medicine, with various health benefits in many cultures. For example, turmeric, which contains curcumin, is known to have anti-inflammatory properties and is considered to help regulate blood sugar, while Javanese turmeric has antioxidant properties and can help digestive function (40). Although many people believe that herbal medicine is beneficial, the use of herbal medicine without proper medical supervision can potentially interact with other medications taken or even delay more effective medical treatment (41, 42).

Using over-the-counter or herbal medications is prevalent among geriatric patients. However, overall medication management remains a challenge. Specifically, patients may be unaware of the possible side effects of herbal medicine and diabetes medications prescribed by medical personnel (43). Therefore, it is essential to inform geriatric patients about the use of these additional medications and carefully monitor their effects on their diabetes control.

In addition, patients often want to try herbal remedies because they are dissatisfied or worried about the side effects of conventional medications, such as nausea, dizziness or digestive problems (41). To ensure diabetes management is carried out holistically and safely, healthcare professionals should discuss with patients all treatment options, including their potential benefits and risks. Therefore, herbal medicine can be considered an adjunct therapy for many geriatric patients. However, this must be done correctly to avoid interference with the primary treatment and to provide optimal benefits in managing T2DM.

## Conclusion

This study shows that geriatric patients with T2DM have various strategies for managing their dietary and medication-taking behaviour, ranging from portion reduction and healthy food choices to spiritual approaches and using herbal medicine. Most of the participants showed awareness and adaptability towards a healthy lifestyle, although there were still challenges, such as old habits, forgetfulness of medication and medication burden. Continuous education and social support are urgently needed to improve the effectiveness of older adults’ self-management in the holistic management of diabetes.

## Figures and Tables

**Table 1:** Socio-demographic characteristics of participants (*N* = 21)

No.	Variable	Category	*n*	%
1	Gender	Male	13	61.9
		Female	8	38.1

2	Age (years)	61 to 65	9	42.9
		66 to 70	6	28.6
		71 to 75	4	19.0
		76 to 81	2	9.5

3	Body mass index (BMI)	Normal weight	14	66.7
		Overweight	6	28.6
		Obese	1	4.8

4	Employment status	Unemployed/retired	17	81.0
		Employed	4	19.0

5	Marital status	Married	17	81.0
		Widowed	4	19.0

6	Monthly income (USD)	0 to 113	9	42.9
		113 to 225	1	4.8
		223 to 338	4	19.0
		339 to 452	7	33.3

7	Distance from home to hospital (km)	0 to 8	15	71.4
		8 to 16	2	9.5
		16 to 24	1	4.8
		24 to 32	3	14.3

8	Companion to the hospital	Alone	11	52.4
		Children	5	23.8
		Wife	3	14.3
		Husband	2	9.5

9	Type of comorbidity	T2DM only	13	61.9
		Hypertension	2	9.5
		Hypertension + Hiperlipidimia	1	4.8
		Hypertension + Gastric disorder	1	4.8
		Heart disease + Gastric disorder	1	4.8
		Heart disease + Neurological disorder	1	4.8
		Gastric disorder	2	9.5

10	Random blood glucose level (mg/dL)	Controlled (< 200 )	14	66.7
		Uncontrolled (≥ 200)	7	33.3

11	Duration of living with T2DM (years)	1 to 5	11	52.4
		5 to 10	8	38.1
		> 10	2	9.5

**Table 2:** Findings of the interview on the topic of dietary patterns

Theme	Sub-theme	Number of participants, *n* (%)
Daily food menu	Reducing the portion of rice and fried foods, replacing them with healthier ones (boiled, grilled, or processed foods from cassava and other tubers)	8 (34.8)
	A combination of rice, vegetables, tempeh, eggs, and a simple side dish	6 (26.1)
	Specific food items and variations (fruits, sea fish, papaya leaves, etc.)	2 (8.7)
	Structured meal schedule (breakfast, lunch, dinner with a specific menu)	4 (17.4)
	Low-portion, simple meals with selected variations (small amount of rice, fruit, and vegetables)	2 (8.7)
	Unchanged diet, still consuming adequate/nutritious foods	1 (4.3)

Controlled food and beverage restrictions	Self-awareness (intentions, personal principles, existing dietary habits, beliefs, awareness of consequences, desire to recover)	10 (47.62)
	Fasting and physical restraint (sleeping, practising patience, engaging in positive activities, and reading the Qur’an)	4 (19.05)
	Reducing or limiting consumption (occasional sugary drinks, small portions, avoiding visual exposure to food, intentional avoidance)	7 (33.33)

**Table 3:** Findings of the interview on the topic of medication behaviour

Theme	Sub-theme	Subcategories/reasons	Number of participants	%
Forgetting to take medication	No incidents of forgetting medication	-	11	52.38

	Forgot to take medication	-	10	47.62
		Out of town/travelling	2	20.00
		Sleeping	2	20.00
		Busy/focus on activities	2	20.00
		Normal/accidental forgetfulness	2	20.00
		Feeling well	1	10.00
		Lazy/careless	1	10.00
		Age-related cognitive decline	1	10.00

Fatigue with medication use	Experiencing boredom/fatigue with medication use	-	11	52.38
		Taking multiple medications or long-term use	7	63.64
		Fear of side effects/complications	2	18.18
		Discomfort due to bitterness	1	9.09
		Feeling well (perceiving no further need for medication)		9.09

	No boredom/fatigue with medication use	-	10	47.62

Non-prescription drug use	Herbal product use (various types)	-	12	57.15
		Turmeric	4	33.30
		Javanese turmeric	3	25.00
		Moringa leaves	2	16.70
		*Minyak angin* (medicated oil)	2	16.70
		Tamarind and turmeric concoctions	2	16.70
		*Jamu godog* (prepared herbal decoction)	2	16.70
		General herbal mix	1	8.30
		Garlic–lime–ginger–vinegar mixture	1	8.30

	No herbal product use	-	9	42.86

## Appendix

### Appendix 1 Semi-Structured Interview Guide

#### A. Dietary Patterns Section

- Could you please describe your typical daily meals?
- How do you manage your cravings for foods or drinks that are restricted due to your diabetes?

#### B. Medication Behaviour Section

- Have you ever forgotten to take your diabetes medication? If yes, what were the reasons?
- Have you ever felt burdened or fatigued by taking your medication? If yes, what causes this feeling?
- Do you take any medications other than those provided by the hospital, such as herbal remedies (jamu)? If yes, could you explain their perceived benefits and why you chose to take them on your own initiative?

Closing Statement for the Participants

Thank you for your willingness to participate in this interview. The information you have provided is valuable for this research. I apologise in advance for any words or actions that may be inappropriate or offensive. Once again, thank you.
